# Mine water as a source of energy: an application in a coalfield in Laciana Valley (León, NW Spain)

**DOI:** 10.1007/s10098-023-02526-y

**Published:** 2023-05-04

**Authors:** A. Matas-Escamilla, R. Álvarez, F. García-Carro, L. Álvarez-Alonso, P. Cienfuegos, J. Menéndez, A. Ordóñez

**Affiliations:** 1 Magna Dea Engineering, Oviedo, Spain; 2 Escuela de Ingeniería de Minas, Energía y Materiales, University of Oviedo, Oviedo, Asturias, Spain; 3 SADIM Engineering, Oviedo, Spain

**Keywords:** Mine water, Geothermal use, Decision-making tool, District heating, Renewable energy

## Abstract

Mine water can be a renewable and economical source of geothermal and hydraulic energy. Nine discharges from closed and flooded coal mines in the Laciana Valley (León, NW Spain) have been studied. Various technologies for the energy use of mine water, as well as the influence of factors such as temperature, the need for water treatment, investment, potential customers and expansion capacity, have been evaluated by means of a decision-making tool. It is concluded that the most advantageous option is an open-loop geothermal system using the waters of a mountain mine, the temperature of which exceeds 14 °C and whose distance to customers is less than 2 km. A technical–economic viability study for a district heating network designed to supply heating and hot water to six public buildings in the nearby town of Villablino is presented. The proposed use of mine water might help areas that have been greatly affected socioeconomically by the closure of the mines and has other advantages compared to conventional energy systems, such as the reduction of CO_2_ emissions.

**Graphical Abstract:**

It showing the advantages of using mine water as an energy source for district heating and a simplified layout.
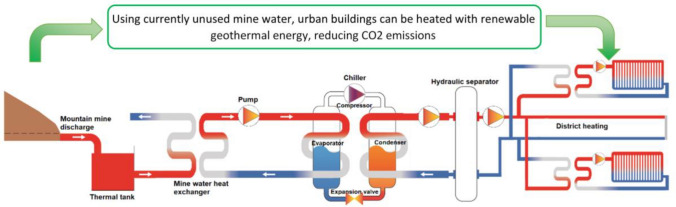

**Supplementary Information:**

The online version contains supplementary material available at 10.1007/s10098-023-02526-y.

## Introduction

Since the Industrial Revolution, fossil fuels have been the dominant energy sources, both in electricity generation and heating. However, in recent years, renewable energy sources have gained importance in order to comply with the guidelines set out in the 2015 Paris Agreement and the goal of zero carbon emissions by 2050. To reach this goal, energy-related global emissions should be reduced to 30% below 2019 levels by 2030, and 75% by 2040 (BloombergNEF 2022). On the other hand, global energy demand is expected to grow by more than a quarter by 2040 and the current situation due to the COVID-19 pandemic and the war in Ukraine has only made the situation more demanding due to the uncertainty of energy supply and the high energy prices. Demand is expected to be achieved by promoting the accelerated development of clean, low-carbon renewable energy sources and improving energy efficiency, as set out in Directive (EU) 2018/2002. An outlook based on the current configuration shows a scenario in which fossil fuel demand slows to a plateau in the 2030s and then falls slightly by 2050, as almost all the growth in energy demand will come from low-emission sources (IEA 2022).

Among these renewable sources, hydroelectric and geothermal are the most suitable systems to obtain energy from mine water, so they are considered in this work.

Hydro-energy can be defined as the energy harnessed from flowing water through a turbine to generate electricity, in such a way that an electric generator converts the mechanical energy of rotation into electric power. A reservoir is required to store water before it moves through the hydraulic turbine. During periods of lower demand, the water is pumped to the upper reservoir and during times of high demand, the water flows back through turbines to produce electricity. The use of hydroelectric generation systems is being promoted throughout the world, considering that water is a renewable, free and abundant energy resource. This type of technology is very well known and proven, since it is over a century old. Over time, there have been relevant developments in its design (structures, components and distribution systems), and hydro-energy has become one of the largest renewable energy sources for electricity generation and energy storage in the world (Marahatta et al. 2022; Kadiyala et al. 2016; Wagner and Mathur 2011). In particular, mine water can be used to generate hydro-power (Jardón et al. 2013) and store energy, for example by means of the underground pumped hydroelectric energy storage systems (UPHS) (Álvarez et al. 2021).

Geothermal energy is that stored in the form of heat below the solid surface of the Earth, which is transmitted from its inner layers and accumulates in rocks and groundwater (Barbier 2002). It is considered a renewable energy (the Earth’s heat is unlimited on a human scale and will be available for future generations) and economical (the investment cost of a geothermal installation for heating is greater to conventional systems, but its maintenance costs are lower and its performance is higher; Self et al. 2013). It is also efficient (it is considered the best for heating and cooling by the US Environmental Protection Agency; US EPA 1997), available (it is continuous throughout the year and accessible in all countries, not depending on external factors) and clean (it allows for reductions in greenhouse gases emissions; Self et al. 2013). Installations that use heat pumps for heating, cooling and the production of Domestic Hot Water (DHW) consume electrical energy for the operation of electric compressors, circulation pumps and building fans, but the emissions are much lower than those of conventional systems (Rosen and Koohi-Fayegh 2017). Additionally, geothermal systems are generally well accepted, particularly in countries with established geothermal industries, and most people are supportive of them, since they are not intrusive and in many cases they are almost invisible or with low impact, due to the compact installations (Sundell and Rämä 2022; Tester et al. 2021; Glassley 2015;). US Environmental Protection Agency (US EPA; 1997) estimated that geothermal heat pumps could reduce energy consumption by up to 72% compared to conventional electrical heating and air conditioning. CO_2_ emission reductions from 15 to 77% were achieved through the use of heat pumps in comparison with residential fossil fuel heating systems (US EPA 1997; Omer 2008). The geothermal potential of mine water, particularly from closed coal mines, is well recognized all over the world, due to its stable and often high temperature, since the coal mines are usually deep and extensive and have a large volume of voids which become a large reservoir when the mine is flooded. Even low-temperature (low enthalpy) mine water resources might provide direct-use geothermal energy for space heating (Preene and Younger 2014). Geothermal is the most common energy use of mine water, particularly at coal mines, and there are applications in several countries in Europe and North America (Banks et al. 2022; Walls et al. 2021; Menéndez et al. 2020; Andrés et al. 2017; Peralta et al. 2015; Watzlaf and Ackman 2006).

Challenges for mine water geothermal energy include mine water quality. Iron sulfides, which are often present in coal deposits, can be exposed and oxidize in mine voids when the mine is active, so when they are flooded, water quality can deteriorate. It has been shown that this leads to the formation of acidic waters, rich in iron and sulfates, or in the presence of carbonates, the waters can be hard and encrusting (Walls et al. 2022, 2021; Younger et al. 2002). The discharging mine water after flooding an underground mine often must be treated, but its quality usually improves gradually over time, according to the effect known as ‘first flush’ (Wolkersdorfer et al. 2022; Younger et al. 2002). These mine water characteristics might compromise its geothermal use, since mine water can be susceptible to mineral precipitation and might corrode or clog heat exchangers or cause environmental impact, but it largely depends on the case (Walls et al. 2021; Loredo et al. 2017; Wolkersdorfer et al. 2008). Mine waters have variable flow and quality, depending on the hydrogeology, the type of mining exploitation and the geological materials involved. Mine water can also be used as a water resource for agricultural, industrial, livestock, environmental or domestic purposes, provided that its hydrochemical characteristics meet the specific requirements demanded. Many mine waters, particularly the drainages from mountain or drift mining, could be used for human consumption without requiring more intensive treatment than usual (Loredo et al. 2017; Ordóñez et al. 2012; Wolkersdorfer et al. 2008).

The circular economy strategy in Europe (EU 2020) proposes to reduce the generation of waste, increase reuse, improve efficiency in the use of water and reduce the emission of greenhouse gases. In particular, the sustainable use of mine water makes it possible to transform what is normally considered waste into a renewable energy resource, giving it a second life, which is of particular interest in old mining areas in decline. The objective of this work is to study the possibilities of energy use of the mine water discharges in the Laciana Valley (León, NW Spain) and to categorize them according to a technical–economic feasibility study, in order to provide a development alternative to a former mining area that is currently economically and demographically disadvantaged.

## Site description

**Fig. 1:**
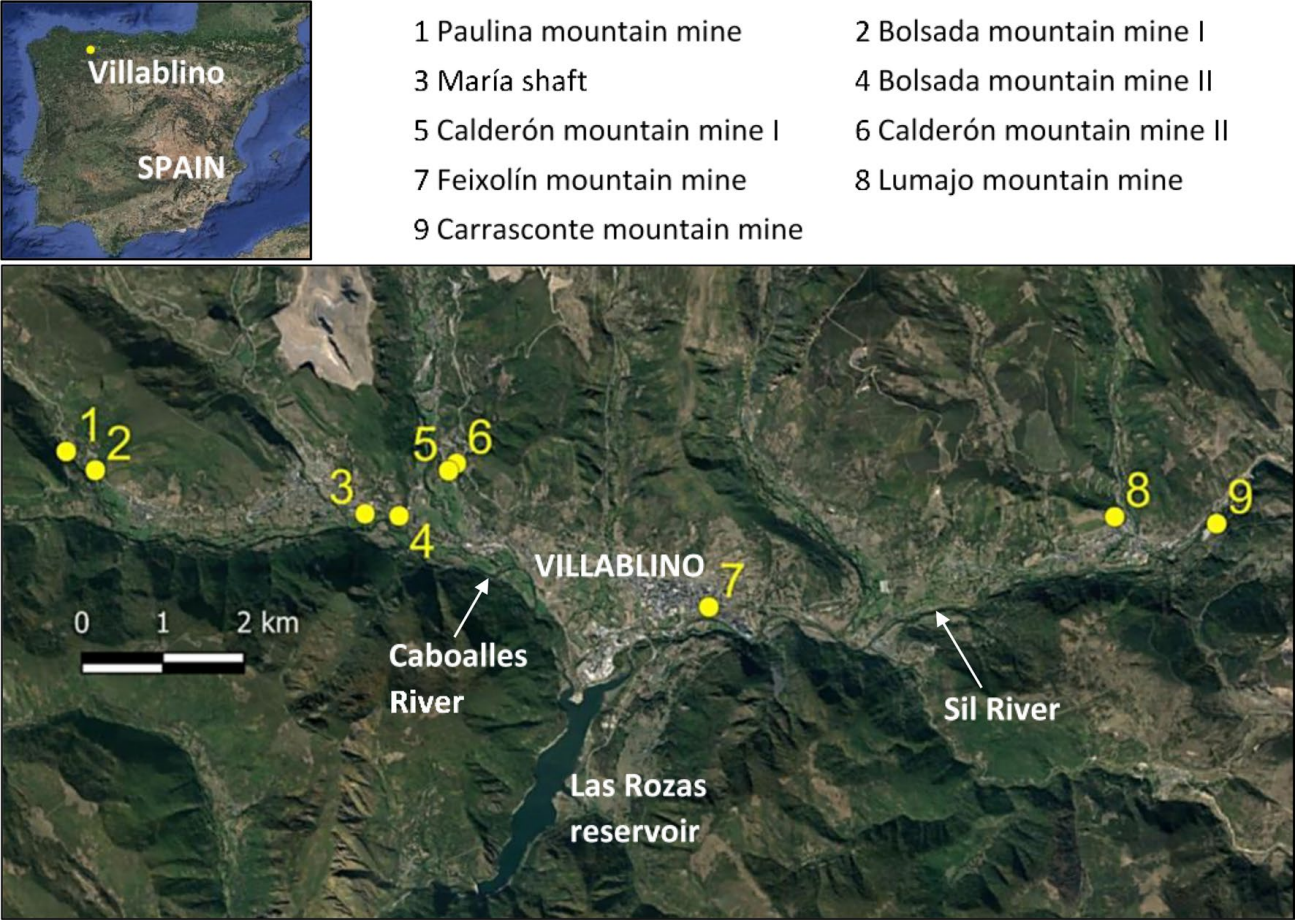
Location of the studied area and mine water discharges (numbers 1 to 9) (Google Earth base image)

The Laciana Valley is located within the León province (NW Spain; Fig. 1), with Villablino being the most important urban area. Coal mining has been the main industry in this area since the beginning of the twentieth century, with up to twelve active mines. “Mountain mining” was first undertaken from the level of the valleys (700 m) to the highest outcrop of the coal seams (1400 m). Subsequently, the exploitation continued through vertical shafts to access the lower layers (underground mining) and open pit mines were also developed. By 2010, all the mines were closed and abandoned and the population has almost halved from the 1990s to the present. Villablino municipality has now around 8,400 inhabitants, and the town (Fig. 1) has 4800 inhabitants.

The study area is located at an altitude of about 1000 m above sea level and its climate is quite temperate, with an average annual temperature of around 7 °C and rainfall about 1100 mm/year. In terms of hydrology, the Sil River is the main watercourse in the area, which joins the Caboalles River at the Rozas reservoir (Fig. 1).

### Geology

The Villablino coalfield comprises Carboniferous (Stephanian B-C age) rocks that unconformably overlie the Narcea antiform in an E-W elongate outcrop of around 120 km^2^. At the eastern and western edges, it partially covers the early Paleozoic sequences of the Cantabrian and West Asturian-Leonese Zones (Lotze 1945). The coal-bearing sequence appears as a NW–SE trending syncline, partially faulted and cut by a thrust on its southern limb. Lithologically, the basin infill is constituted by a 2500–3000 m thick sequence of detrital sediments deposited in a continental sedimentary environment that includes conglomerates, sandstones (mainly litharenites), slates and coal seams (Knight and Álvarez-Vázquez 2021). There are 24 economically exploited coal seams ranging from 0.4 to 5.8 m thick. In the lower half of this sequence, there are igneous bodies of acidic-intermediate composition with porphyritic textures up to 15 m thick, which have generated natural coking phenomena when intruded near a coal layer. Coal rank includes both bituminous A and anthracite C (ISO 11760) with vitrinite reflectance between 1.12 and 4.24% (Colmenero et al. 2008).

### Hydrogeology

Most of the sedimentary rocks in the studied area have low permeability (Ordóñez et al. 2012). However, mining activities increase permeability by creating voids and inducing fracturing, so groundwater flows preferentially through open spaces, fractures and zones of decompression. When a mine is active, any permeable layer intercepted by the mining works, will induce a flow of water to the mining voids, so they must be drained in order to continue exploitation. In mountain mining, the drainage is produced by gravity, through the lowest gallery, creating a gradual decline of the water table to the mining front of advance. Springs are replaced by mine adits, from which mine water is discharged naturally. In contrast, in underground mines pumping is maintained to drain the mines and to keep all the mining works dry (Ordóñez et al. 2012). Once the mining activity finishes, if pumping is stopped, the gradual flooding of the mine voids will occur, phenomenon known as “groundwater rebound” (Gandy and Younger 2007). Mining voids filled with water behave like a karstic aquifer or “underground mining reservoir,” which recharges with rainfall and discharges typically through the lowest mine adit or a permeable layer hydraulically connected to the flooded mine voids (Álvarez et al. 2018). Uncontrolled mine water discharges are often undesirable, particularly if they are close to towns and/or have environmental impacts for surface water and ecology, but maintenance of the pumping is unaffordable when the mining companies are no longer active (Jardón et al. 2013; Álvarez et al. 2016). Pumping is often restored and adjusted to maintain a permanent flood level in this created mining reservoir for security and environmental reasons, which can be regulated and used as a water or energy resource, particularly geothermal (Álvarez et al. 2021; Menéndez et al. 2019; Hall et al. 2011). In the Laciana Valley, the waters coming out from the mine adits are generally discharged into the closest watercourse. In this case, the studied mines have been closed more than a decade ago and no pumping is maintained. The mines studied in this work are mostly mountain mines, so they are drained by gravity flow. This means that the rainfall infiltrates and after a short period of residence (4 to 10 days; Jardón 2010) inside the mine, it is discharged from the lowest mine adit, at the height of the valley and they end up in the close watercourses (Caboalles and Sil Rivers; Fig. 1). Similar to a spring, the flow of these discharges depends on the rainfall rate and is variable along the year, but they never run dry. Since the catchment area of these mines is not extensive, the water flows are not very high (maximum around 0.1 m^3^ s^−1^ in wet periods and mean values around half of that number).

## Materials and methods

### Hydrochemistry

In this work, the waters discharging from eight mountain mines (Fig. 1) and one underground mine (sampling point #3 at María shaft) were studied to characterize them for energy use. Five sampling campaigns were undertaken seasonally in 2017–2018. Samples were taken right at the entrance of the mine adits, and water flow was measured using a flow meter when it was possible and by means of the volumetric or the float methods in other case. Parameters such as pH, temperature, electrical conductivity and turbidity were measured in situ over the 5 campaigns by means of HANNA multi-parameter probes. Water hardness was determined through a JBL test. Langelier and Ryznar indices (Rafferty 2000) were calculated in order to estimate the corrosive or fouling character of the sampled waters. Multi-elemental analysis was performed by ICP-AES at the ALS Global Laboratory, in samples previously acidified taken in one campaign in 2017.

### Geothermal potential of mine water

The thermal potential P (W) that can be extracted from a fluid (in this case mine water) is given by the formula (Ochsner 2008): (Eq. 1)

1 $$P{\mkern 1mu} = {\mkern 1mu} F \cdot \Delta T \cdot {\text{SH}} \cdot \rho$$

where F is the water flow (m^3^ s^−1^), ΔT is the temperature difference extracted from the water across a heat pump (ºC), SH is the water specific heat (4186.8 J kg^−1^ ºC^−1^), and ρ is the water density (1000 kg m^−3^). It is obvious from the equation that the higher the water flow, the higher the thermal power of the resource. It is easy to think that the higher the mine water temperature, the lower the necessary thermal difference in the chillers and, therefore, the better performance the system will have. However, the performance depends also on the temperature range the heat pump is designed for and the amount of electricity used should be taken into account. Generally, the greater the temperature difference through which the heat pump works, the less efficient the heat pump will be, since a higher temperature differential implies a lower amount of heat pumped or a higher amount of power required by the heat pump (Banks 2012). Notwithstanding, the approach in this paper considers geothermal potential based on flow rate as in Eq. 1.

### Decision-making

In order to evaluate the options for energy use, a multi-attribute decision-making method (MADM) was applied, considering the 9 studied waters and 4 energy use alternatives:

i) Open-loop geothermal use: Mine water passes directly through a heat exchanger and transmits its thermal energy to a secondary circuit that feeds the evaporator of the chiller.
ii) Closed-loop geothermal use: A circuit of pipes is introduced inside the mine, with thermal exchange between the mine water and the fluid flowing inside the circuit. This network of pipes constitutes the evaporator circuit of the chiller, saving an intermediate heat exchanger with respect to the previous configuration (Banks et al. 2017).
iii) Hydroelectric use: A minimum flow of water and a sufficient hydraulic head (> 20 m) are required. In this case, it is possible to use the height difference between galleries within the mine shafts or to channel the water that comes to the surface to a suitable place where a hydroelectric power plant can be built.
iv) Hydroelectric use by means of microturbines: The mine water can be dammed and generate a low hydraulic head (5–10 m). Microturbines can generate electrical energy by joining a conduit or stream of water with reduced flow (Gatte and Kadhim 2012).

**Fig. 2:**
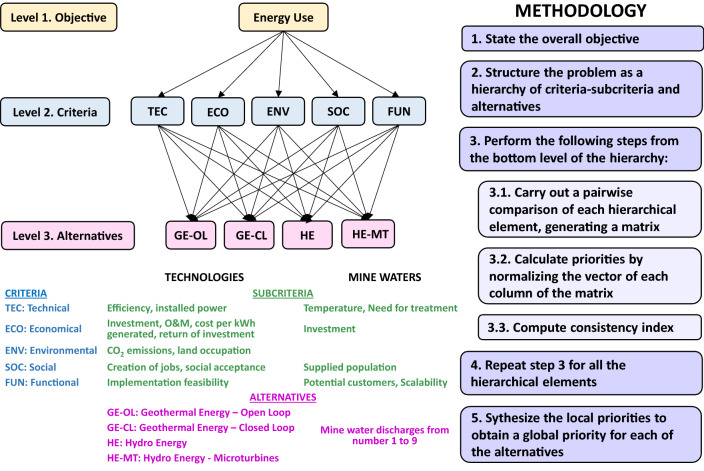
Synthetic methodology of the used Analytical Hierarchy Procedure (Kablan 2004; Saaty 1990)

The Analytical Hierarchy Procedure (AHP) was chosen because it is the most widely used subjective weighting method in studies of energy system alternatives (Pohekar and Ramachandran 2004). A second evaluation method was incorporated, so that the score obtained in the AHP is transformed through a fuzzy analysis following the trend of current evaluation methods that seek flexibility and the incorporation of uncertainty management (Strantzali and Aravosis 2016). The methodology followed is summarized in Fig. 2. Criteria established for the different technologies and the alternatives of mine waters were: technical, economical, environmental, social and functional. These criteria were compared with each other and weighted, according to the AHP methodology, so that the weights are consistent (Matas 2021). To compare the different technologies, the efficiency of geothermal energy to generate electricity was considered, although it is not specifically its purpose, as stated by Evans et al. (2009).

### Design

An energy use is proposed through a district heating network that provides heating and/or sanitary hot water to several public buildings in Villablino: the Town Hall, the House of Culture, a municipal sports center, a primary school, a secondary school and a health center. Firstly, a study of the demand of these clients was carried out to find out their needs. Subsequently, the possibilities of the available mine waters to meet this demand and the need of a storage system to guarantee supply throughout the year, were estimated. The hot water generation system was designed, as well as the hydraulic circuit to transport the water from the mining facilities to the clients. The hydraulic circuit was modeled using the EPANET 2.0 software. The hydraulic dimensioning (e.g., head loss and friction) was carried out applying the Darcy-Weisbach and Colebrook-White formulas (Matas 2021). Finally, the remodeling of the clients’ building control systems was designed to be able to adopt the new energy supply system.

### Economical study

The process followed to carry out the economic analysis of this project is as follows: First, the benefits of the sale of geothermal energy to customers are calculated as a product of the energy demanded by the system (kWh) that would be covered by the geothermal installation and the price per kWh. Next, the operation and maintenance costs (including electrical energy and overall costs of the system components) are calculated using the equation:

2 $${\text{O}}\& {\text{M cost }}\left( {euro {\text{ year}}^{{ - {1}}} } \right) \, = { 65},{792}\;\;\;P\; + {6},{334}.{3}$$

(Menéndez et al. 2020), where *P* is the power in MW.

Finally, the 25-year NPV, as well as the initial investment are calculated and they are both compared taking into account external subsidy scenarios.

## Results and discussion

### Hydrochemistry

**Fig. 3:**
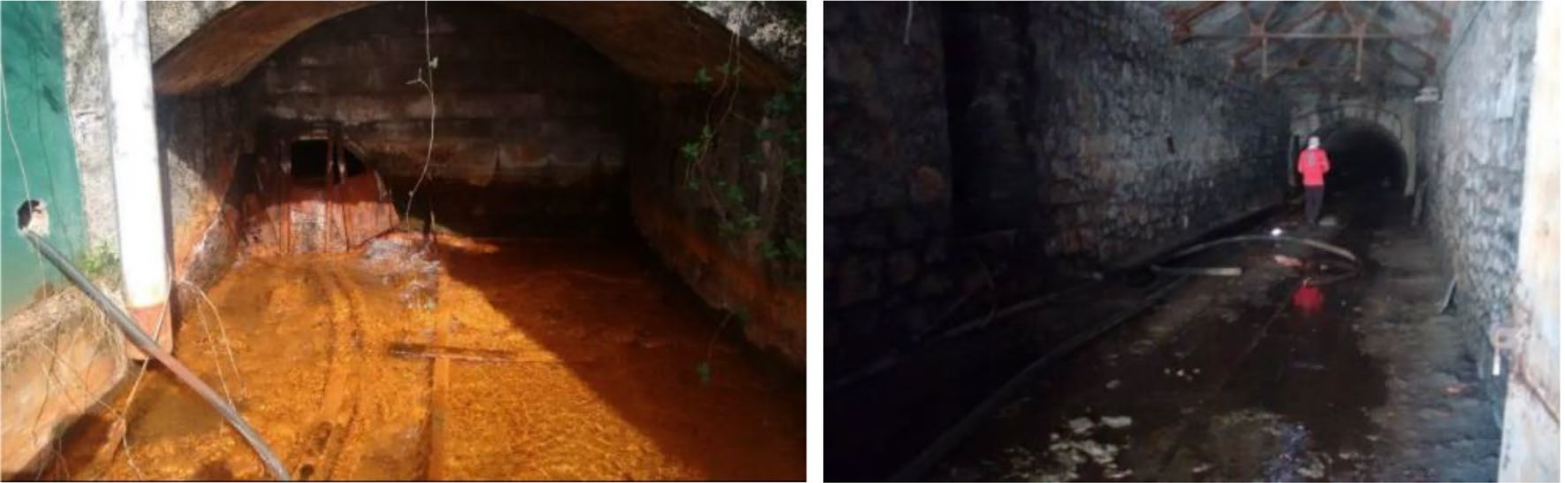
Mine water discharge points at Carrasconte (left) and Feixolin (right) mines

**Fig. 4:**
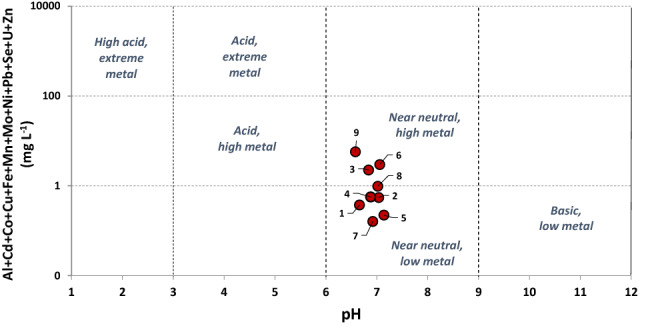
Classification of the studied waters according to diagram of Fichlin et al. (1992)

Table 1 shows the arithmetic mean values of the parameters measured in situ in the studied mine waters (Fig. 3), as well as the chemical results (the whole data are shown in the spreadsheet of Supplementary Information). The highest temperatures are found in the sampling points 4, 5 and 7 (Calderón and Feixolín mines), whereas the coldest water is that coming from Paulina mine, at the highest sampling point (#1 at 1145 m a.s.l.). Although they are warmer in the summer months, the temperature is quite stable in all the sampled waters throughout the year. All of them are circumneutral, reaching their highest pH values in winter, possibly due to the dilution effect of the increased rainfall rate. The lowest pH value was measured in Paulina mine water in the summer (6.36). This is in agreement with the alkalinity due to carbonates, which is strong, excepting in the waters from Paulina and Bolsada mines, which are moderately alkaline. Waters from Calderón and Feixolín mines are those with the lowest turbidity, but they have high TDS values. Electrical conductivity is above 1 mS cm^−1^ in the waters sampled at the points #4, #5, #6 and #9. All the waters are very hard (> 180 mg L^−1^ CaCO_3_; Akram and Rehman 2018). According to the classification of Fichlin et al. (1992; Fig. 4), the sampled mine waters are all near neutral and they have low metal concentrations, excepting waters #3, #6 and #9, which have high metal contents (particularly Fe).

**Table 1:** Average values found in sampled waters (UTM coordinates ETRS89 ZONE 29; the number of asterisks indicates a greater degree of scaling or corrosive character of the water, according to the values of the Langelier and Ryznar indices)

Mine water	*X* coord	*Y* coord	Temp	pH	El. Cond	TDS	Hardness	Ca	Mg	Al	Mn	Fe	Thermal	Langelier	Ryznar	Character	
	UTM	UTM	°C	uds	µS cm^−1^	mg L^−1^	mg L^−1^ CaCO_3_	mg L^−1^	mg L^−1^	µg L^−1^	µg L^−1^	µg L^−1^	Pot. (kW)	index	index	Fouling	Corrosive
1	711,231	4,759,273	9.8	6.6	399	435	249	60.5	25.3	6.74	19.1	329	733	−1.0	8.6		***
2	711,589	4,759,044	10.0	7.1	281	317	214	51.7	24.1	17.17	99.3	387	209	−0.8	8.7		***
3	716,038	4,759,124	13.9	6.8	890	938	458	88.1	67.8	9.02	439	1,799	209	−0.1	6.9		*
4	715,331	4,758,484	12.2	6.8	1,045	887	587	104	68.6	7.16	93.3	456	1,151	−0.1	7.1		**
5	716,038	4,759,124	14.4	7.2	1,394	1156	823	137	166	8.30	6.37	186	1,072	0.4	6.3	*	*
6	715,941	4,759,039	14.7	7.0	1,934	1,447	957	164	184	10.51	292	2,637	1,047	0.5	6.0	*	*
7	719,146	4,757,362	14.6	7.0	798	748	405	88.7	68.2	10.75	26.7	103	1,001	−0.1	7.1		**
8	724,144	4,758,473	12.9	7.0	528	568	311	62.3	38.8	8.05	220	738	733	−0.3	7.6		**
9	725,400	4,758,388	12.5	6.6	1,664	1,566	988	168	161	21.45	463	5,114	1,256	0.1	6.4	*	*

The quality of the water used in a geothermal system is decisive and can seriously affects its efficiency, causing scaling or corrosion (Walls et al. 2021). Mine water that passes through the heat exchangers in geothermal installations must not have a corrosive or fouling character to guarantee its durability and efficiency (Loredo et al. 2017; Teng et al. 2016). According to the obtained Langelier and Ryznar indices (Table 1), the sampled waters have little or no fouling nature, but are mostly considered corrosive. The waters from the María (#3), Calderón (#5 and #6) and Carrasconte (#9) mines are only slightly corrosive, whereas the waters from Paulina (#1) and Bolsada (#2) mines are highly corrosive.

Concentrations in Fe and Mn are elevated in some cases, particularly in the waters from the María mine (#3) and the Carrasconte mountain mine (#9; Fig. 3 left). On the other hand, Fe and Mn concentrations in the waters arising from the Calderón mine I (#5) and Feixolín mine (#7; Fig. 3 right) are below the Spanish drinking water requirements.

### Geothermal potential of mine water

The water from the Calderón mine I (#5) has an average flow of 51.2 l s^−1^ and an average temperature of 14.4ºC. According to (Eq. 1) and considering a thermal difference of 5ºC, which is usually considered for common chillers, the thermal potential of this mine water is 1072 kW (Table 1). The highest thermal potential was found for the waters from the Carrasconte (#9), Bolsada (#4), Calderón I and II (#5 and #6) and Feixolín (#7) mines. Among these waters, the best choices are #5 and #7, since waters from #4 and #9 have a relative low temperature and waters from #6 and #9 have a high metal content.

### Decision-making

When applying the AHP to the studied area, the four alternatives for energy use stated in Sect. "Decision-making" were valued, according to the established criteria. Hydroelectric use (options iii & iv) was prioritized in terms of the technical criteria, but it is also the most expensive. Regarding the environmental criteria, geothermal uses (options i & ii) generate more CO_2_ emissions, but they imply less land occupation. As for social criteria, hydroelectric use (option iii) is the best scored in terms of job creation, but it is less socially accepted than the rest of alternatives. Finally, open-circuit geothermal use (option i) is the best weighted in terms of implementation feasibility. One of the most important advantages of open geothermal circuits is that the water source remains at the same temperature throughout the year (Boahen et al. 2017). Considering all the priority vectors, the most advantageous technology to be applied in the Laciana Valley is the use of open-circuit geothermal energy (option i), followed by hydroelectric use by means of microturbines (option iv). According to the already stated criteria, the available mine waters were also evaluated. As can be seen in Table 2, the criteria with the best scores in the analysis of the mine water alternatives were the economic and the functional, followed by the technical and finally the social one. The best scored sub-criteria are the temperature and the investment. With the application of the AHP described, the mine water with the best score is that of the Feixolín mine (#7), due to its relatively high temperature, good quality and short distance to the Villablino town (with more potential consumers).

**Table 2:** Criteria and weightings given to the studied mine waters in the application of the Analytical Hierarchy Procedure

Criteria	Score	Sub-criteria	Score
Technical	2	Temperature	3
		Need for treatment	2
Economical	3	Investment	3
Social	1	Supplied population	2
Functional	3	Potential customers	2
		Scalability	2

Mine water	Rating
1	2.5
2	3.1
3	7.8
4	3.6
5	7.5
6	6.3
7	8.1
8	5.5
9	4.6

### Design

The daily thermal energy demand was studied for each building considered for the district heating network, taking into account the different spaces and specific routines. These buildings currently use biomass, gasoil and coal boilers. Table 3 shows the maximum hourly demand of each month of the year of the six public buildings. The health center has the highest needs, since it is the only one that has demand throughout the day. The system as a whole will demand 2426.8 kWh in the hour of greatest demand, considering that all customers are connected to the network, and the consumption peak is between 7:00 and 8:00 on winter time. The minimum demand of the system takes place in summer (July and August) with 61.9 kWh required to heat the health center. Table 3 also shows the annual demand of each building, calculated considering the hourly demand along each day of the year. Total demand covered with this geothermal system is 4040 MWh_t_ year^−1^ and this would avoid the emission of 1160 or 1402 t CO_2_ per year, in comparison with gasoil and coal boilers systems, respectively (Spanish Government 2016). Although CO_2_ emissions are actually released during the burning of wood biomass, it is considered as a carbon–neutral energy source by the EU (2022). For this calculation, it was applied an equivalence of CO_2_ emissions from conventional systems to generate the thermal energy, but without considering the electrical energy consumption of the installation. The installation of renewable sources (for example, photovoltaic solar) for the supply of electrical energy in a self-consumption mode, the cost of which would also be subsidized, would allow a more complete reduction of emissions.

**Table 3:** Maximum hourly demand (kWh) in each month of the year and annual demand (kWh year^−1^) of the buildings of the district heating

	Town hall	House of culture	Municipal sports center	Primary school	Secondary school	Health center	Total
January	127.2	107.2	250.0	176.7	734.5	1,031.2	2,426.8
February	127.2	107.2	250.0	176.7	734.5	1,031.2	2,426.8
March	127.2	107.2	250.0	176.7	514.2	1,031.2	2,206.4
April	63.6	53.6	125.0	88.4	367.3	773.4	1,471.2
May	0.0	0.0	50.0	0.0	73.5	515.6	639.1
June	0.0	0.0	50.0	0.0	73.5	309.4	432.8
July	0.0	0.0	50.0	0.0	0.0	309.4	359.4
August	0.0	0.0	50.0	0.0	0.0	309.4	359.4
September	0.0	0.0	50.0	0.0	73.5	515.6	639.1
October	63.6	53.6	125.0	88.4	367.3	773.4	1,471.2
November	127.2	107.2	250.0	176.7	587.6	1,031.2	2,279.9
December	127.2	107.2	250.0	176.7	734.5	1,031.2	2,426.8
Annual demand	137,376	144,687	319,200	238,545	958,523	2,242,613	4,040,943

In the installation of heating and cooling systems using mine water from closed coal mines, low-enthalpy geothermal plants equipped with heat exchangers and chillers are generally installed in the mine facilities (Menéndez et al. 2019, 2020). The supply of heating to the buildings is carried out by means of district heating systems using insulated pipes. The temperature of the supplied water depends on the existing heating system in the building but old heating systems are normally kept as backup systems, so they can work in case of problems or breakdowns. As an example, an open loop geothermal plant was installed in the Asturian Central Coal Basin (NW Spain) to provide space heating and cooling to a Hospital located 2 km from the mine, through an exchanger and three chillers. However, to ensure the supply of thermal energy, the Hospital’s thermal plant also includes three gas boilers as a backup system (Menéndez et al. 2020). In this study, it was not intended to replace the heating systems that currently exist inside the buildings, but to keep them as a backup. Geothermal energy will only be used when the demand can be supplied with it, but the current conventional heating systems would remain so that they can be used in peak times, when the customers demand more energy than geothermal energy can supply. The system can be thus optimized, based on the existing installations, to meet reasonably the customer demand without trigger costs due to an elevated installed power. Maximum demand occurs during a few hours throughout the year (in January and December), so it is not economically feasible to implement a system that guarantees 100% demand coverage. It is then considered as an objective that the proposed system can cover 98% of the demand, leaving out the 168 h with the highest demand of the 8760 total hours per year. This is achieved stablishing an energy supply of 1900 kWh, which would not meet the demand between 6:45 and 8:15 on weekdays in the winter months. In addition, in order to avoid operating problems at low load, a minimum demand of 500 kWh (only not reached in the summer months), is established for the system to come into operation. This supposes a considerable economic saving with respect to installing equipment to cover 100% of the demand.

**Fig. 5:**
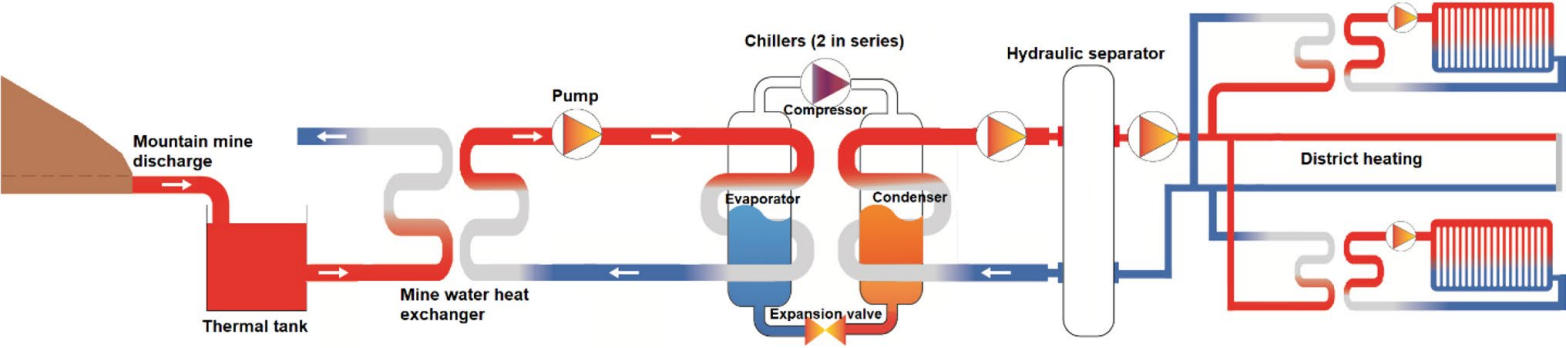
Schematic design of the main components of the geothermal system

The generation system of the proposed district heating integrates the following main components (Fig. 5):

1. *Thermal tank*: reservoir that accumulates mine water (maintaining its temperature) in order to regulate the system.

2. *Mine water heat exchanger:* a separate element of the circuit that transmits heat from the mine water to the evaporator circuit, which transports mains water (less aggressive) so the evaporators of the chillers are protected by keeping independent circuits. In this case, a shell and tube heat exchanger was selected due to its large commercial implementation, its lower initial costs, as well as its reliability (McGeorge 2012).

3. *Chillers*: they are equipment that take heat from one medium and transfer it to another through a thermodynamic Carnot cycle. In this case, heat is taken from the water in the evaporator circuit, which goes from 12°C (temperature at the entrance of the chiller) to 7°C, to deliver it to the water in the condenser circuit, and by means of a secondary circuit, water increases its temperature from 60 to 75°C.

The hot water generation system includes a mine water heat exchanger, chillers, hydraulic pumps, a degasser, an expansion system and a thermal tank (Fig. 5).

As described above, the maximum power of the generation system was defined (1900 kWh), as well as the chillers’ inlet and outlet temperatures (75–60 °C on the condenser side and 7–12 °C on the evaporator side), and also the general layout of the chillers (two units in series and countercurrent). Then, it is possible to obtain the flow rates that maximize the performance of the chillers (COP) through the Carnot diagram for the selected refrigerant (R1234-ZE) and taking into account the operating points and thermodynamic and enthalpy parameters of the system (method described in full in Matas 2021). The flow rates are 60.7 L s^−1^ in the evaporator and 31.2 L s^−1^ in the condenser. This results in a COP of 3.20 for the first chiller and 2.61 for the second one.

The heat loss due to *conductions* of a district heating network is one of the fundamental elements since they must transport the hot water to each of the clients without allowing the fluid they transport to lose its temperature. Therefore, these ducts must be thermo-insulated using polyurethane foam (Dalla-Rosa et al. 2011). In this case, plastic pipes are chosen for the distribution circuit, because the water temperatures are in the right range for them, their ease of installation and their economic advantages (Italsan 2016). The pipes with their heat insulation have been included in the cost of the design (initial investment).

Hydraulic calculations were made for the four circuits in the system related to: distribution, evaporation, condensation and mine water. First circuit connects the generation room to the customers with pre-insulated polypropylene pipes whose diameters were calculated to be between DN90 and DN250. Last three circuits run entirely in the generation room and are composed of pre-insulated metallic piping with a nominal diameter of 8 inches. The building control systems must be specifically remodeled and adapted, by dimensioning the equipment and specifying the connections between the new system and the existing one. Sizing of the exchange substations was considered, since the head loss of the substations is an input to the hydraulic calculation of the circuit.

**Fig. 6:**
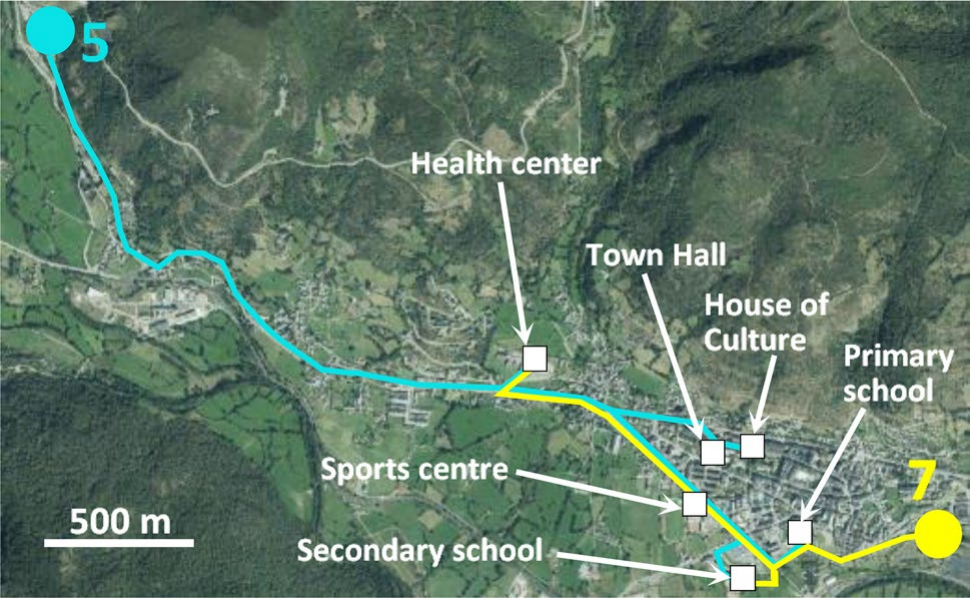
Proposed network using water from the Calderón mine I (#5) and from the Feixolín mine (#7)

The network using water from the Calderón mine I (#5) is 10.9 km, whereas it is 5.8 km if water from the Feixolín mine (#7) is used (Fig. 6). The hydraulic circuit was modeled with EPANET 2.0 in order to determine the adequate water flows, pipes and pumps, as well as the load losses.

### Economical study

The initial investment is very high, mainly due to the cost of pipes and trenches. This value increases with distance of the mine water discharge from customers and with the diameter of the pipes. Distances of the Calderon mine I (#5) and Feixolín mine (#7) to the urban center are 3.31 and 0.95 km, respectively. Total length of conductions (of various sizes) from the Calderón mine I to the six buildings would be 10.9 km, what implies a cost of 3.12 M€. In contrast, the total conductions from the Feixolín mine add up to 5.8 km, with a cost of 1.07 M€. This fact is so relevant that if the water from the Calderón mine I is used, the system is not economically profitable (negative NPV) (Matas 2021), so it is preferred to use the water from the Feixolín mine, with a similar temperature and flow but much closer to the customers. To reduce this cost, customers who are less than 2 km away should be prioritized (Menéndez et al. 2020). This in turn will allow the use of smaller diameters of the pipes because pressure losses will be reduced.

Tables 4, 5 show the results of the economic study carried out from both the entrepreneur and the client’s perspectives, respectively. From the promoter's point of view, there is a scant difference between the operating and maintenance costs and the benefits produced by the sale of thermal energy. Although it depends on the negotiations with the clients, the margins are narrow. Taking into account that the profit from the sale of geothermal energy is 202,047 € per year and the O&M cost given by Eq. 2, a profit of 65,470 € year^−1^ is obtained, which annualized over 25 years with a discount rate of 8% amounts to a profit of 79,975 €.

**Table 4:** Main economic values from the promoter’s point of view. Data from Menéndez et al. (2020) and Matas (2021)

Implementation cost (€)	Demand covered (kWh year^−1^)	Geothermal price (€ kWh^−1^)	Geothermal profit (€ year^−1^)	Subsidy (%)	25-year NPV (€)
3,374,089	4,040,943	0.05	202,047	80	79,975

**Table 5:** Main economic values from the customer’s point of view (case of the secondary school). Data from Menéndez et al. (2020), Matas (2021) and Clickgasoil (2022)

Heating system	Heat demand (kWh year^−1^)	Consumption (L)	Gasoil price (€ L^−1^)	Geothermal price (€ kWh^−1^)	Heating cost (€ year^−1^)	Profit (€ year^−1^)
Gasoil	958,523	30,928	1.61	-	49,856	0
Geothermal	958,523	-	-	0,05	47,926	1,930

**Fig. 7:**
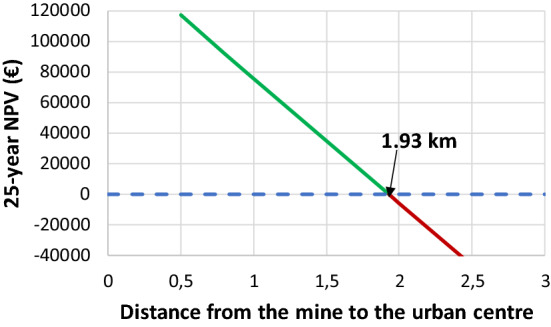
Variation of the NPV with the distance between the mine and the urban center

The European Commission is promoting initiatives that help mitigate the social consequences of the low-carbon transition in coal-mining regions. The use of closed coal mines to produce renewable thermal energy can receive subsidies to boost the local economy. In addition, in the surroundings of the mines there are not usually large consumption centers and what is proposed here is a autochthonous energy source that would reduce energy dependence. It is necessary to increase the profit margins from the sale of energy through premiums that favor this renewable energy or through subsidies that greatly reduce the initial investment to undertake (Matas 2021). In this case, using the water from the Feixolín mine, the system can be profitable for a promoter who sells the thermal energy to the clients, considering a subsidy scenario of the initial investment. In this case, the whole system, with all its elements and the necessary connections to the existing equipment in each of the buildings considered, has an implementation cost of 3.37 M€. Considering a subsidy of 80% (usual for Next Generation subsidies) and a selling price of thermal energy of 0.05 € per kWh_t_, the 25-year NPV analysis of the return on investment yields a positive value of 79,975€. In this scenario, the use of a mine water discharge start being profitable at a distance of 1.93 km from the urban center, where the customers are located (Fig. 7). In this situation, no mine in Valle Laciana is profitable without a subsidy, which is consistent with previous studies (Menéndez et al. 2020). Other active installations for energy use of mine water, such as the one mentioned above in Asturias, have also received significant subsidies from European funds to start up.

Notwithstanding, from the client’s perspective, it is profitable to replace a gasoil or a gas boiler by a mine water geothermal system. Gasoil and coal costs have risen sharply recently, while the cost of geothermal energy has remained more stable (Richter 2022). Therefore, customers who switch from gasoil to geothermal systems can save about 2000€ per year.

## Conclusions

Flooded mines constitute an interesting option for geothermal and hydraulic energy generation in many regions of the world. Mine water can be used for heating using geothermal chiller systems, reducing the emission of greenhouse gases and energy dependence. In particular, in the Laciana Valley (León, NW Spain), coal mining has been carried out for most of the twentieth century, but the massive closure of the mines in this century has had a serious economic and social impact on the area. Groundwater rebound due to the cessation of pumping in the closed mines has caused their flooding, so that mine water discharges on the surface occur. In this work, a technical and economic analysis of the potential use of some of these mine water discharges for heating of public buildings is presented.

The potential of the discharges was evaluated by means of a decision-making method. In terms of the type of technology, the most suitable is shown to be an open-loop geothermal system. Regarding the characterization of the available mine waters, although some factors such as the water temperature or the water quantity and quality are relevant, the decisive factor appears to be the distance from the water source to the potential customers. A district heating network that could provide heating or sanitary hot water to six cultural, sports and health buildings in the town of Villablino was designed, incorporating components such as chillers, mine water exchanger, thermal tank or conduction circuits, being the latter particularly costly. Thus, the most profitable system is that using the water from the closest mine to the town. A geothermal plant with an investment cost of 3.37 M€ and an annual production of thermal energy of 4040 MWh is designed to supply heating services. The results show that the pipe length (distance from the mines to the users) and the joint demand for thermal energy are critical to optimize the efficiency and profitability.

In this case, like other active analogous installations, in order to make the system profitable, from the promoter’s perspective, it is necessary a subsidy to reduce the initial investment, whereas the customers get large savings replacing conventional non-renewable systems by geothermal ones. Other potential clients could later join this network, which would result in more benefits. In addition, other possible uses for mine water could be explored, like using it to generate hydraulic energy or to provide greater regulation capacity to the nearby Las Rozas reservoir. In any case, a reduction in CO_2_ emissions, compared to conventional systems, would be achieved.

The use of mine water makes it possible to value a resource that is generally considered as a residue. Quality and quantity of these mine water discharges must be controlled periodically in the future, as well as paying attention to possible potential users whose supply could be profitable in an energy scenario as variable as the current one. Waters of the closed mines at Laciana valley are coming out naturally, so their geothermal use would mean a triple benefit for an old coal mining area: environmental, economic and social, and this is the case of many other mining districts.

## Supplementary Information

Below is the link to the electronic supplementary material.

Supplementary file1 (XLSX 22 KB)

## Data Availability

Enquiries about data availability should be directed to the authors.
